# Performance and usability of Cepheid GeneXpert HIV-1 qualitative and quantitative assay in Kenya

**DOI:** 10.1371/journal.pone.0213865

**Published:** 2019-03-22

**Authors:** Priska Bwana, Joshua Ageng’o, Matilu Mwau

**Affiliations:** Kenya Medical Research Institute, Busia, Kenya; University of Cape Town Faculty of Health Sciences, SOUTH AFRICA

## Abstract

**Background:**

In Kenya, access to early infant diagnosis and viral load monitoring services for HIV patients on ART is significantly hampered by sample transportation challenges and long turnaround times. Near patient care testing technologies have the potential to obviate such constraints. The Cepheid GeneXpert was launched in 2010 as a TB assay and in 2014 as a potential point of care HIV viral load assay. Whereas it is widely is used for TB in Kenya, its utility for HIV testing has not been evaluated.

**Objective:**

To investigate the performance and usability characteristics of the GeneXpert HIV-1 qualitative and quantitative assay.

**Methods:**

This was a cross sectional study among 911 HIV Exposed infants and 310 HIV positive adults. Existing machines used for routine TB diagnosis were used in this study. The diagnostic accuracy of the qualitative assay was assessed using Roche CAP/CTM while the quantitative assay was assessed using with Abbott m2000 as the reference assays respectively. Statistical analysis was done using Stata/MP Version 14 for Mac. Concordance values and misclassification were calculated at the clinical cutoff of 1000 cp/ml.

**Results:**

The sensitivity, specificity and accuracy of the GeneXpert HIV-1 qualitative assay were 99.23% (95% CI 97.24–99.90%), 98.91% (95% CI 97.76–99.55%) and 99.00% respectively. For the quantitative assay, they were 92.50% (95% CI 79.61–98.43%), 100.00% and 97.00% respectively. All 30 (100%) users reported that the GeneXpert machine was easy to use, workflow was simple and TB diagnosis was not negatively affected. In our hands, the median turn-around time for an individual qualitative and quantitative test was 90 minutes. A total of 58 (4.34%) errors and 28 (2.10%) invalid outcomes were experienced; 44 (3.29%) tests did not run to completion due to power outages.

**Conclusion:**

GeneXpert HIV-1 qualitative and quantitative assay is an accurate test for the diagnosis of HIV in infants and for viral load monitoring. At the point of care, the GeneXpert machine’s simple work flow, ease of use and short test turnaround time present the potential to improve access to HIV testing and viral load monitoring. To integrate HIV diagnosis into the existing GeneXpert platforms for TB Diagnosis, there is need to scale up the infrastructure and to change the way work is done.

## Introduction

By 2015, 1.5 million (4.1%) of the 36.7 million people living with HIV worldwide were Kenyan[1, 2]. Both early infant diagnosis and viral load testing, which are essential for monitoring HIV treatment care and are critical to achieving the UNAIDS 90-90-90 targets by 2020 [3]. Kenya has managed to reduce the prevalence of HIV from 7.4% in 2007[4] to 5.9% in 2015 [2]. Highly active antiretroviral therapy (HAART) is widely accessible and by 2017, treatment coverage was at 75%[5].

Despite having 9 reference HIV testing labs, EID and VL testing coverage is only around 76% and 98% respectively[5]. Sample transportation over long difficult roads and prolonged turnaround time to results hamper wider access to these services[6, 7]. Although the hub and spoke model[8, 9] has been implemented to improve sample networking, the hubs themselves are not testing labs and the turnaround time still remains a challenge. Loss to follow up is significant. Near patient and point of care testing technologies have the potential to obviate these challenges.

WHO recently approved several point of care assays for HIV diagnostics, including Cepheid Xpert HIV-1 Qual for HIV qualitative testing[10, 11] using whole blood or dried blood spots (DBS)[12] and Cepheid XpertHIV-1 Viral Load for HIV quantitative testing [13–15] using plasma, both using one fully integrated cartridge[16]. These near patient assays, have reported encouraging performance in recent studies[17, 18]. Among other test platforms [15, 19, 20] still undergoing development, is the GeneXpert Omni, a portable, battery-operated, wireless, and web-enabled diagnostic test that can transmit test and instrument information in real time[21].

In Kenya, both Alere q HIV 1/2 Detect and Xpert HIV-1 qualitative assays have been approved for Early Infant diagnosis. Cepheid Xpert MTB/RIF is also widely used for TB diagnosis. However, neither Alere q HIV 1/2 Viral Load nor Cepheid Xpert HIV-1 Viral Load has been approved for viral load quantification. Additionally, the utility of already existing GeneXpert platforms for HIV testing alongside TB diagnostics has not been evaluated.

This evaluation was intended to determine the accuracy and ease of use of the Xpert HIV-1 qualitative and quantitative assay (Cepheid, Sunnyvale, California, United States) in field evaluations.

## Methodology

### Study design

All the GeneXpert machines used for this study were already in situ and routinely used for TB diagnosis. At study inception, it was noted that all machines were in use during the day for TB diagnosis, since all the facilities that had them were hubs within a testing network. It was determined that HIV testing was best done beginning late afternoon. Study personnel, though experienced users, were trained for this purpose. Both the qualitative and the quantitative studies of the performance of the GeneXpert platform were cross sectional evaluations of samples obtained from facilities across the country. The usability data was collected through structured questionnaires.

In order to assess the performance of the Xpert HIV-1 quantitative assay on GeneXpert platform, 100 plasma samples were tested on both the platform and on the Abbott m2000 assay and the results compared.

To assess how different sample types performed against each other on the GeneXpert platform, 107 comparisons were made between plasma and whole blood, and 84 comparisons between plasma and DBS.

To assess the qualitative performance of the Xpert HIV-1 qualitative assay, 911 infant DBS samples were tested against Roche CAP/CTM.

#### HIV qualitative analysis

In field sites, two DBS filter papers were collected from infants as previously described [22]. One of the filter papers was processed and tested on the GeneXpert platform, according to the manufacturer’s instructions. Briefly, from each filter paper, one dried blood spot was cut out and dropped into a vial containing proprietary buffer and incubated in an Eppendorf thermomixer for 15 minutes at 56° C while rotating at 500 rpm. The contents of the vial were then added into the Xpert HIV-1 Qual test cartridge and loaded onto the GeneXpert machine. Results were observed and recorded after 90 minutes. The second DBS filter paper was shipped to the reference lab and tested on the Roche CAP/CTM platform according to manufacturer’s instructions as previously described [23]. The results were recorded as HIV detected or HIV not detected.

#### Quantitative performance evaluation

For viral load estimation on the GeneXpert platform a total of 1.2 ml of plasma was added into the Xpert HIV-1 Viral Load cartridge using a calibrated pipette. The cartridge was closed and loaded onto the machine. Test results were observed and recorded after 90 minutes. The limit of detection was 40 cp/ml and the clinical cut off was 1000 cp/ml. For comparison, viral load estimation was done on the Abbott m2000 using plasma according to manufacturer’s instructions. Briefly, 0.6ml of plasma was transferred into a reaction tube, which was then inserted into the Abbott m2000sp machine worktable. Automated sample extraction was conducted using m2000 version 2.0 open mode 0.6ml RNA HIV-1 assay protocol. The products were retrieved for RNA amplification and quantification, on the Abbott m2000rt platform. Test results were observed and recorded after 5 hours and the limit of detection was 40 cp/ml. The clinical cut off was 1000 cp/ml.

#### Suitability of different sample types for use on GeneXpert platform

From each venous blood draw, whole blood, DBS and plasma was prepared and tested on GeneXpert platform.

Plasma was processed as for performance evaluation.

For DBS, one spot was cut out of the filter paper, dropped into an Eppendorf tube and 1.2 ml of 20 mM Tris HCl was added. The tube was incubated in a thermo mixer for 15 minutes at 56° C while rotating at 500 rpm. All the contents of the Eppendorf tube were poured into the sample chamber of the Xpert HIV-1 Viral Load cartridge which was then loaded onto the machine. Test results were retrieved after 90 minutes. The limit of detection was 40 cp/ml and the clinical cut off was 1000 cp/ml.

For whole blood, 0.75 ml of 20 mM Tris HCl buffer was first added onto the Xpert HIV-1 Viral Load cartridge. 100 μl of Whole blood sample was then added without delay. The cartridge was then handled according to the manufacturer’s instructions. The limit of detection was 40 cp/ml and the clinical cut off was 1000 cp/ml.

#### Usability evaluation

All study personnel received training on the assays being evaluated and were certified in their use. A structured questionnaire capturing steps to results, amount of waste generated, ease of use, errors and overall opinion of the technology was filled in by each personnel based on their experience on the machine. The usability data was collected by 30 GeneXpert platform users in Meru, Kakamega, Homa Bay and Siaya County referral Hospitals, as well as Yala, Pap Onditi, Ndhiwa and Kibera Sub County hospitals.

### Ethical considerations

This study was reviewed and approved by the Kenya Medical Research Institute’s Scientific Ethical Review Unit (KEMRI/SERU Protocol No. 2657). Since this was a research study, written informed consent was requested for study participation from every participant. In the case of minors, written informed consent was obtained from parents or guardians. The study was conducted in accordance with the ethical standards of the Helsinki Declaration of 1975, as revised in 2000.

### Statistical analysis

Viral load data was transformed into log_10_ cp/ml. Statistical analysis was performed using Stata/MP Version 14 for Mac. Accuracy was estimated using a 2 x 2 table at a cut off of 1000cp/ml. Descriptive statistics were used to compare misclassification between platforms and sample types. Bland Altman comparison was conducted to assess the limits of agreement and the mean bias. Pearson Correlation Coefficient (r^2^) was used to assess the linear fit for paired readings of log transformed viral load copies between platforms and between sample types. Simple descriptive statistics was used to report usability data.

## Results

### Performance characteristics of the GeneXpert quantitative assay

The concordance between the GeneXpert and Abbott m2000 assay at the clinical cut off of 1000cp/ml is depicted in Table 1. All of the 37 samples testing >1000cp/ml on the Abbott m2000 assay tested >1000 cp/ml on the GeneXpert platform. Three of the 63 samples which tested <1000cp/ml on the Abbott m2000 assay tested >1000 cp/ml on the GeneXpert assay. Therefore, the overall concordance was 97.00% with an upward misclassification of 7.50%. There was 0% downward misclassification (Table 1).

**Table 1:** Concordance between the Xpert HIV-1 and Abbott m2000 viral load assay at a clinical cut off of 1000cp/ml using plasma.

	**Abbott Viral Load assay results**		
**Xpert Viral Load assay Results**	**>1000cp/ml**	**<1000cp/ml**	**Total**
**>1000cp/ml**	37 (92.50%)	3 (7.50%)	40 (40.00%)
**<1000cp/ml**	0 (0.00%)	60 (100.00%)	60 (60.00%)
**Total**	37 (37.00%)	63 (63.00%)	100 (100.00%)

**Fig 1:**
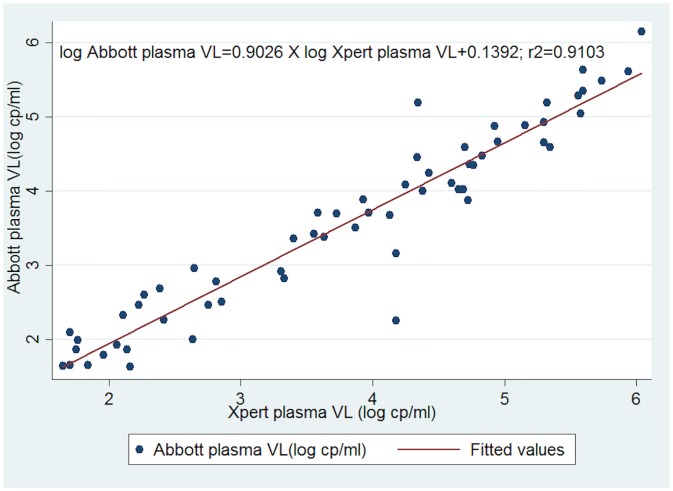
Scatter plot of log transformed viral loads of Abbott m2000 against GeneXpert plasma test.

A scatter plot of log transformed values indicated an excellent fit with a slope of 0.9026. Linear regression revealed a statistically significant positive relationship between the two assays with a Pearson Correlation Coefficient (r^2^) of 0.9103 (95% CI 0.8280–0.9771, p<0.05, Fig 1).

Bland Altman analysis indicated a positive mean difference of 0.331 logs (-0.558 to 1.220 logs). We did not observe any systematic trend from lower and higher values.

**Fig 2:**
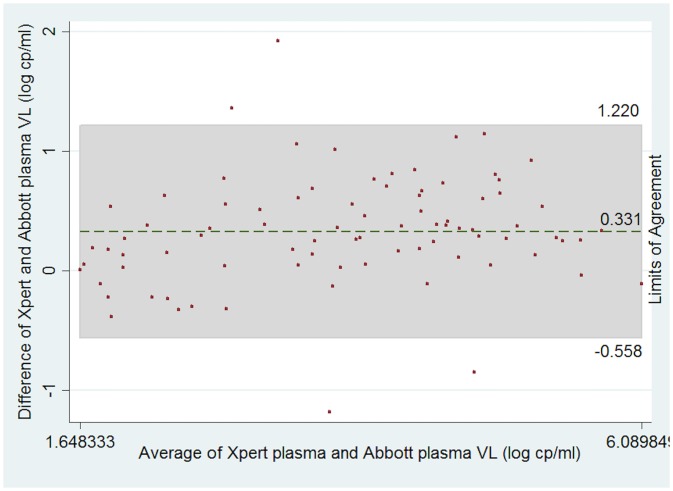
A Bland-Altman analysis of log transformed viral loads of GeneXpert and Abbott m2000 plasma assays.

This is shown in Fig 2.

### Performance of different samples types on the GeneXpert platform

In a comparison of paired samples, all the 107 whole blood samples had detectable viral loads while only 91 plasma samples had detectable viral loads. At the clinical cut off of 1000cp/ml, 90 (84.11%) whole blood samples showed concordance with plasma while 5 (12.20%) showed down misclassification and 12 (18.18%) up misclassification (Table 2).

**Table 2:** Concordance between whole blood and plasma on the Xpert HIV-1 viral load assay at a clinical cut off of 1000cp/ml.

	**Plasma Xpert Viral Load assay results**		
**Whole blood Xpert Viral Load assay results**	**>1000cp/ml**	**<1000cp/ml**	**Total**
**>1000cp/ml**	36 (87.80%)	12 (18.18%)	48 (44.86%)
**<1000cp/ml**	5 (12.20%)	54 (81.82%)	59 (55.14%)
**Total**	41(100.00%)	66 (100.00%)	107(100.00%)

Linear regression between the sample two types yielded a Pearson Correlation Coefficient (r^2^) of 0.7501 (0.5291–0.7108, p<0.05).

Only 84 paired DBS and plasma samples were successfully compared. At the clinical cut off of 1000cp/ml, 53 (63.10%) DBS samples showed concordance with plasma while 31 (36.90%) showed down misclassification. Up misclassification was not observed.

### Qualitative evaluation of GeneXpert HIV-1 assay

A total of 911 HIV-exposed infants (HEI, median age = 1.5 months), were successfully recruited for the qualitative evaluation of GeneXpert HIV-1 Assay. A total of 901 DBS samples were successfully collected and tested on both the Xpert and Roche CAP/CTM platforms.

The sensitivity of the Xpert HIV-1 Qual assay was 97.36% (95% CI 94.63–98.93%) while the specificity was 99.69% (95% CI 98.87–99.96%). The concordance was therefore 99.00%. Table 3 below summarizes the performance characteristics of Xpert HIV -1 Qual assay.

**Table 3:** Concordance between Roche CAP/CTM and the Xpert HIV-1 Qual assay using DBS.

	**CAP/CTM Qual assay results**		
**Xpert Qual assay Results**	**Positive**	**Negative**	**Total**
**Positive**	258 (97.36%)	2 (0.31%)	260 (28.86%)
**Negative**	7 (2.64%)	634 (99.69%)	641 (71.14%)
**Total**	265 (100.00%)	636 (100.00%)	901(100.00%)

### Usability characteristics

Of the 1555 qualitative and quantitative tests conducted on GeneXpert platform (including repeats and duplicates), 1436 (92.35%) tests passed. Sixteen (16, 1.03%) tests failed due to machine user errors, 38 (2.44%) due to hardware errors and 8 (0.51%) due to temperature. A further 28 (1.80%) tests gave invalid results while 29 (1.86%) tests failed due to power outage. The overall error rate was 7.65%.

All the 30 (100%) users reported that the machine was easy to use, the work flow was simple, and the waste generated was little. There were five steps from sample collection to results. The turn-around time for each test was approximately 90 minutes. The GeneXpert machine’s footprint was less than 4 square feet for the 16 module and less than 1 square foot for the 4 module. The machine was not rechargeable but had GSM capability.

## Discussion

In the attempt to achieve UNAIDS 90:90:90 goals, Kenya has come up with innovative approaches aimed at reaching underserved populations, including the hub and spoke model of laboratory networking, and the adoption of molecular point of care testing technologies.

Recent WHO guidelines recommend technologies that deliver a “sample in/result out” service at the point of care[24]. They should perform well using not only whole blood but also plasma and dried blood. For HIV molecular diagnosis, only a few such technologies are available.

Many countries have developed innovative ways of integrating HIV, TB and malaria programs in order to increase efficiency of resource usage and to improve the quality of health of patients. The GeneXpert platform is widely available in Kenya for TB diagnostics. It can help integrate both TB and HIV diagnosis at the point of care, but before our study this potential had not been explored. In some countries, GeneXpert devices introduced for TB diagnostics are underutilized; that spare capacity can be leveraged for HIV diagnostics. GeneXpert is the primary tool for TB diagnostics in Kenya and in our study, we found that the machines were busy throughout the day, often dealing with backlogs. To prevent disruption to service delivery, we negotiated to test for HIV in the late afternoons and evenings. We opine that health care workers who use GeneXpert for TB diagnosis at the current time would be amenable to testing for HIV using the same platform seamlessly if the national policy so required. Furthermore, if GeneXpert Omni proves to be similarly accurate in evaluations, then this technology has the potential to lower level health care facilities for service delivery. The impact of such interventions should be assessed periodically.

In Kenya, the sample type of choice for early infant diagnosis is DBS. Even in near patient settings, DBS remains an attractive solution, mainly due to the complexity of sample collection in infants and also for the reason that many facilities in rural Kenya have embraced the hub and spoke model for service delivery. Using DBS, the concordance of GeneXpert HIV-1 qualitative assay was 99.00%, a finding corroborated by other studies[25]. This level of accuracy is very high and when viewed together with the challenges of transporting samples to central labs indicates that the GeneXpert qualitative assay has a significant role in EID testing in Kenya at the point of care.

The GeneXpert quantitative assay performed poorly on dried blood spots in our hands. However, for quantitative testing at the point of care and in near patient settings, DBS is an inconvenient sample type, and is unlikely to come into common usage, and therefore we consider the poor performance of the assay on DBS to be of minimal significance at the program level. The fact that the GeneXpert HIV-1 quantitative assay performed sub-optimally on whole blood is of much greater concern at the point of care, considering that blood is the easiest and most convenient sample type. The reason for this poor performance is unclear to us, but is supported by other studies[26]. However, when plasma samples were used instead, the GeneXpert HIV-1 quantitative assay was comparable in performance to the Abbott m2000 HIV-1/2assay. The preparation of plasma in most health facilities in Kenya is a routine activity, and the role of the assay in viral load testing at the point of care is therefore clear. That said, further assay optimization using whole blood will be necessary to minimize resource wastage.

Current clinical guidelines use a cutoff of 1000 cp/ml for clinical decision-making. With plasma, a concordance of 97.00% was observed at that cutoff. Studies conducted on similar platforms and sample type have recorded varied performance with the concordance values ranging between 91.32%-100%[25–30]. Our findings also agree with those observed by WHO[31].

In the GeneXpert qualitative assay, upward misclassification of 0.31% and downward misclassification of 2.64% was observed. This misclassification is quite low considering the intended use at the point of care.

For the quantitative assay using plasma, upward misclassification was 3(3.00%) at the clinical cut off of 1000cp/ml. There was no downward misclassification. This low misclassification rate compares favorably with other studies[26]. and indicates that GeneXpert can be used interchangeably with Abbott m2000 without anticipation of clinically relevant bias.

When whole blood was compared with plasma on GeneXpert at a cut off of 1000cp/ml, 5 (4.67%) downward misclassifications and 12 (11.21%) upward misclassifications were observed. Clinically, this high upward misclassification rate would lead to more patients being identified as potential treatment failures, requiring adherence counseling and further plasma based VL testing for confirmation.

When DBS was compared with plasma on GeneXpert at a cut off of 1000cp/ml, 31(36.90%) down misclassification was observed while upward misclassification was not reported. If DBS were to be used as the sample of choice, many patients would be would be identified as suppressed when they actually are not. This would unnecessarily expose them to complications related to high viral load. The suboptimal performance of whole blood and dried blood spots suggests that further optimization of the assay is necessary to improve performance. The positive bias of the quantitative GeneXpert assay has been reported in other studies[26].

The overall test failure rate on the platform was 7.65%. This rate is comparable to similar studies[32]. A majority of errors were hardware related, at 2.44%, followed by errors due to power outage, at 1.86%. Of note, user errors were uncommon, at 1.03%. This is important especially since user training can be capital intensive for some assays in the market. Temperature-related errors accounted for only 0.51% of all, implying that the technology is quite appropriate for sub-Saharan Africa, where temperatures can be as high as 40° C. Whereas errors experienced did not affect the overall performance of the respective assays, because all failed tests were repeated, they did lead to loss of cartridges and time, which are important considerations in resource limited settings. During testing, 28 (2.10%) tests gave invalid results on both the Xpert Qual and **Viral Load** assay. These invalid results may have been related to quality control during the manufacturing process or insufficient sample volumes. With strict adherence to good manufacturing practise by the manufacturer, and sufficient sample volumes, these errors may be reduced or eliminated altogether.

The main strengths of this study lie in the fact that we assessed the usability and quantitative and qualitative performance of GeneXpert simultaneously in the context of existing platforms used for TB diagnostics.

## Study limitations

The most important limitation of this study was that data collection was not directly observed by the investigators, the quality of the work could not be guaranteed. However, the technicians were experienced and were well trained.

The test devices did not have inbuilt power and went off during power outages. This resulted in 44 (3.29%) tests not running to completion. There is a definite need for a power back up for use in the event of power outage to reduce wastage and turnaround time to results.

## Conclusions

In this study, we have demonstrated that at the point of care, the GeneXpert assay shows excellent performance, has a simple work flow, is easy to use and has a short test turnaround time. Scale-up of the Xpert TB assay and the existing infrastructure and changing the way HIV and TB diagnostics work is done could serve as an implementation platform for the Xpert HIV VL and EID assays. Therefore, the GeneXpert assay can be used as a point of care assay for viral load estimation in resource limited settings.

## Supporting information

S1 File GeneXpert qualitative study questionnaire. (PDF)

S2 File GeneXpert quantitative study questionnaire. (PDF)

S3 File GeneXpert usability assessment form. (PDF)
