# Supplementary material for: Performance and usability of Cepheid GeneXpert HIV-1 qualitative and quantitative assay in Kenya
Source: PLoS One. 2019 Mar 22;14(3):e0213865. doi: 10.1371/journal.pone.0213865 (PMC6430374; doi:10.1371/journal.pone.0213865)
Supplement: S1 File — (PDF) [file pone.0213865.s001.pdf]

GENEXPERT QUALITATIVE STUDY QUESTIONNAIRE

Date………………………….

Study Identification number………………………….

Age (days/week/months) ……………………… Point of entry: …………………………

Gender: Male ( ) Female ( )

Infant Prophylaxis: …………………………………

Mother HIV status.................................... Mother ART regime ……….………………

Name of Operator: ……………………..

Cartridge Batch: ……………………….

DBS POC Result: …………………….

Other Comments (Errors, reason for repeat/invalid test, etc):

……………………………………………………………………………………………….

……………………………………………………………………………………………….

……………………………………………………………………………………………….

……………………………………………………………………………………………….
