# Supplementary material for: Performance and usability of Cepheid GeneXpert HIV-1 qualitative and quantitative assay in Kenya
Source: PLoS One. 2019 Mar 22;14(3):e0213865. doi: 10.1371/journal.pone.0213865 (PMC6430374; doi:10.1371/journal.pone.0213865)
Supplement: S2 File — (PDF) [file pone.0213865.s002.pdf]

GENEXPERT QUANTITATIVE STUDY QUESTIONNAIRE

Date………………………….

Study Identification number………………………….

Date of birth ………………………………   Age (years) ………………………

Gender: Male ( )   Female ( )

HIV Status: …………………   Are you on antiretroviral therapy: …………………

Which ones? ....   For how long have you been on ART? :………………

GeneXpert

Name of Operator: ……………………..

Cartridge Batch: ……………………….

Plasma Result …………….. Whole blood Result …………………….   DBS Result: ……………………

Other Comments (Errors, reason for repeat/invalid test, etc):

…………………………………………………………………………………………………….

…………………………………………………………………………………………………….

…………………………………………………………………………………………………….

…………………………………………………………………………………………………….
