# Supplementary material for: Performance and usability of Cepheid GeneXpert HIV-1 qualitative and quantitative assay in Kenya
Source: PLoS One. 2019 Mar 22;14(3):e0213865. doi: 10.1371/journal.pone.0213865 (PMC6430374; doi:10.1371/journal.pone.0213865)
Supplement: S3 File — (PDF) [file pone.0213865.s003.pdf]

GENEXPERT USABILITY ASSESSMENT FORM

**Name of Test:**	**Name of Facility:**		
	YES	NO	COMMENT
Are the test instructions clear and simple?			
Does the package indicate the storage conditions?			
What are the recommended storage conditions?			
What is the shelf life of the test cartridge?			
How many steps to results (Pre analytical-post analytical) are there?			
How long does the test take in minutes to result?			
Is it easy to interpret the test results?			
What happens when power fluctuates?			
Does the machine require a power back up?			
Which types of errors have you experienced?			
How would you rate the overall ease of use?			
Would you recommend the technology for use?			
How do you rate the individual test packaging?<br>Poor ☐ Tolerable ☐ Good ☐ Very good ☐			
How would you rate the training needs?<br>None ☐ Simple ☐ Moderate ☐ Extensive ☐			
How much waste is generated (relatively)?<br>Too much ☐ Very little ☐ Moderate ☐			
What would you say is the space requirement for this machine?<br>Little, ☐ Moderate, ☐ Large ☐			
Other comments?			
